# Potential of Zeolite and Algae in Biomass Immobilization

**DOI:** 10.1155/2018/6563196

**Published:** 2018-12-12

**Authors:** Seyed Amirebrahim Emami Moghaddam, Razif Harun, Mohd Noriznan Mokhtar, Rabitah Zakaria

**Affiliations:** ^1^Department of Chemical and Environmental Engineering, Universiti Putra Malaysia, Serdang, 43400 Selangor, Malaysia; ^2^Department of Process and Food Engineering, Universiti Putra Malaysia, Serdang, 43400 Selangor, Malaysia

## Abstract

The interest in utilizing algae for wastewater treatment has been increased due to many advantages. Algae-wastewater treatment system offers a cost-efficient and environmentally friendly alternative to conventional treatment processes such as electrocoagulation and flocculation. In this biosystem, algae can assimilate nutrients in the wastewater for their growth and simultaneously capture the carbon dioxide from the atmosphere during photosynthesis resulting in a decrease in the greenhouse gaseousness. Furthermore, the algal biomass obtained from the treatment process could be further converted to produce high value-added products. However, the recovery of free suspended algae from the treated effluent is one of the most important challenges during the treatment process as the current methods such as centrifugation and filtration are faced with the high cost. Immobilization of algae is a suitable approach to overcome the harvesting issue. However, there are some drawbacks with the common immobilization carriers such as alginate and polyacrylamide related to low stability and toxicity, respectively. Hence, it is necessary to apply a new carrier without the mentioned problems. One of the carriers that can be a suitable candidate for the immobilization is zeolite. To date, various types of zeolite have been used for the immobilization of cells of bacteria and yeast. If there is any possibility to apply them for the immobilization of algae, it needs to be considered in further studies. This article reviews cell immobilization technique, biomass immobilization onto zeolites, and algal immobilization with their applications. Furthermore, the potential application of zeolite as an ideal carrier for algal immobilization has been discussed.

## 1. Introduction

Water is the basic element of life on earth. The demand for water in the entire world is growing fast for the domestic, agricultural, and industrial activities. However, the availability and quality of water resources face severe threats due to the industrialization and rapid economic development producing a huge amount of wastewater. This wastewater contains organics, suspended solids, and hazardous materials such as heavy metals which are not biodegradable. These materials tend to accumulate in organisms and potentially cause severe contamination and diseases due to poor water management [1, 2]. Hence, wastewater treatment is essential to prevent deterioration of the environment and solve the issue of water shortage and health.

Wastewater treatment consists of removing or decreasing a number of hazardous substances such as chemicals and biological pollutants [3]. The selection of the treatment approaches is strongly influenced by their characteristics and compositions. Various wastewater treatment approaches have been used such as chemical, physical, and biological approaches [3]. Advanced oxidation, electrocoagulation, and flocculation are the most common methods of physical and chemical treatment whereas suspended or activated sludge process is an example of the biological treatment that is widely applied [4]. Although these conventional methods have successfully treated various wastewater sources, they have some limitations. The physical and chemical methods face the problems of high-energy requirements, incomplete removal of heavy metals, generation of secondary pollutants, complex operation, and high cost [4–6], and the biological method deals with the problems of the easy washout and low biomass concentration [4].

The use of microalgae to treat wastewater is currently of global interest due to its advantages. On the one hand, microalgal cells have the ability to uptake nutrients such as phosphorus, nitrogen, and ammonium as well as heavy metals to reduce BOD in wastewater [7–10] and also simultaneously capture the carbon dioxide from the atmosphere during photosynthesis decreasing the greenhouse gaseousness [11, 12]; on the other hand, the wastewater can be considered as a cheaper nutrient source for the growth of microalgae. However, at the current state, the biological nutrient removal technologies, including the use of microalgae, have not been competitive in the wastewater industries. The main issue contributes to the high cost of recovery of the treated effluent and biomass using current dewatering methods such as centrifugation and filtration [11]. To overcome the issue, the existing cell suspended method can be replaced with the immobilization method. The advantages of immobilization over the suspended method have directed researchers to discover in detail the system [3, 4, 13, 14]. Not only can cell immobilization simplify the separation process, but it also offers other advantages such as higher cell density, higher productivity, better cell stability, and biomass recirculation [3, 4, 13, 15, 16]. One of the most important parts in the immobilization technique is selecting a suitable carrier. As the common carriers such as alginate and polyacrylamide face some drawbacks like low stability and toxicity, respectively [13, 17], finding and using a new carrier with ideal characteristics (i.e., nontoxic, stable, and cheap) can solve existing problems. One of the ideal candidates applied for the immobilization is zeolite. Zeolites are inorganic materials that are resistant to microbial degradation and also cost-effective [4, 18]. The application of zeolites is in agriculture, catalysis, building industries, energy, and treatment of water streams [1, 19–45]. In addition to these common applications, they have been used as a carrier for biomass immobilization [5, 15, 46–55] and applied in different areas such as production of high-value products [15, 46, 47, 49, 50, 53] and metal/nonmetal removal [5, 48, 51, 52, 54, 55]. Various kinds of microorganisms have been used for the immobilization onto zeolites such as bacteria [5, 15, 49–54] and yeast [46, 47].

This article reviews the immobilization technique, biomass immobilization onto zeolites as special carriers, and algal immobilization. Furthermore, the potential application of zeolite as an ideal carrier for algal immobilization has been discussed.

## 2. Immobilization

Immobilization is defined as a process of cell attachment and/or cell inclusion to or into a carrier volume as a support [13, 56]. Cell immobilization has a number of advantages over suspended cells shown in Table 1. Immobilized systems have been applied in various applications such as the production of noncontaminant energy [57, 58], wastewater bioremediation [13], toxicity measurement [58, 59], metabolite production [58, 59], and stock culture management [3].

### 2.1. Types of Immobilization

The process of microorganism immobilization can be done by various mechanisms, including adsorption, covalent bonding, cross-linking, encapsulation, and entrapment in a matrix [3, 4, 13, 18, 50, 59–65].  Table 2 shows various types of immobilization method along with their advantages and disadvantages. According to Eroglu et al. 2015 and Godlewska-Żyłkiewicz et al. 2003, the most common immobilization methods are cell attachment and cell entrapment [3, 64]. In immobilization via adsorption, the attachment is done without using intermediate molecules while, in the covalent attachment, the “bridge-molecule” is used for functionalization of the support [63]. In the entrapment method, cells are captured into a three-dimensional gel lattice. This kind of supports can be either natural or synthetic [3, 59, 60, 65]. The method is often used for capturing microorganism in suspended solution. Due to the porous structure of polymers, different kinds of metabolites can be easily diffused through the matrices [4, 18].

### 2.2. Operational Modes of Immobilization

The immobilization process can be done in two operational modes: passive and active [13, 57]. The passive mode is based on the natural ability of the microorganism to attach to the natural or synthetic carriers (solid or gel-like surfaces) and grow on them [4, 13, 57, 59]. The mechanisms for the carrier attachment involve physical (electrostatic and hydrophobic interactions) and chemical (covalent bond formation) [4, 13]. The most common electrostatic interactions involved in the initial stage of adsorption are ionic and hydrogen bonding [4]. In contrast, in the active mode, there is not any dependence on the natural ability of cells for the attachments [13] and the mechanisms for the carrier attachment are covalent bonding, cross-linking, and inclusion of cells into the carrier [4, 13, 57]. In addition, the carrier type is either synthetic or natural [13, 57].

### 2.3. Ideal Carrier for Immobilization

One of the important parts in the immobilization process is selecting a suitable carrier. According to Vasilieva et al. 2016 and Das and Adholeya 2015, there are two types of carriers that can be used for cell immobilization: natural and synthetic [4, 13]. The ideal carrier for cell immobilization has to fulfill some characteristics such as porous structure, light weight, being inert, nonbiodegradable in the test condition, and nontoxic, no inhibition, and allowing mass transfer. Also, the carrier should have high stability, including chemical, mechanical, and biological, be environmentally friendly and cheap, and provide an irregular, rough surface for colonization [4, 13, 66, 67]. To date, various supports have been used in cell immobilization, including organic and inorganic supports. Organic supports have higher absorptivity and larger forms of reaction groups like amino, hydroxyl, and carboxyl than inorganic supports [4]. Alginate, agar, carrageenan, polyacrylamide (PAM), and polyvinyl alcohol (PVA) are some of the examples of organic carriers. Another form of carrier materials is inorganic such as clay, activated charcoal, and zeolite. These materials have some characteristics, including resistance to microbial degradation, suitable thermostability performance, and cost-effectiveness [4, 18].

## 3. Biomass Immobilization onto Zeolites

### 3.1. Zeolite

**Figure 1:**
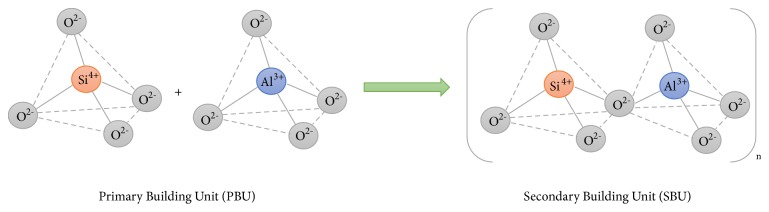
Primary and secondary building units of natural zeolite (adapted from [69]).

Zeolites are inorganic materials whose structures contain Primary and Secondary Building Units, PBU and SBU, respectively. PBU builds up from SiO_4_ and AlO_4_ tetrahedra and they are connected via oxygen ions to form SBU. The SBU are then linked into a three-dimensional crystalline structure and form a honeycomb shape [41, 68, 69]. Due to the substitution of Si^4+^ by Al^3+^, the charge of the framework is negative, which is balanced by monovalent or divalent cations located together with water [38, 69]. As a result, because of surface negative charge, zeolites are capable of exchanging ions with an external medium [38, 69]; hence, they can be used for metal ion removal in water environments.  Figure 1 shows primary and secondary building units of natural zeolite.

There are three types of zeolite characterized as natural, modified, and synthetic. Up to now, over forty types of natural and one hundred types of synthetic or modified zeolite have been reported [30]. Natural zeolites are of common forms such as clinoptilolite, mordenite, etc., but the most abundant one is clinoptilolite [30, 38]. The natural zeolites are known in the world for their characteristics, including ion exchange capacities, high porosity, large specific surface area, and low cost [19, 26, 45, 51]. However, the problem with the natural zeolites is that their ion-exchange capacity is relatively low [19]. Hence, some modifications are needed to solve this issue, where modified and synthetic zeolites are the candidates. Modified zeolites, for the most part, are obtained via either acid or base treatment and surfactant modification methods. Meanwhile, synthetic zeolites are obtained from some materials like clay minerals, kaolin, waste materials, etc. [30]. From the point of view of cation exchange capacity and sorption performance, both modified and synthetic zeolites have higher capacity and performance than the natural zeolite [30]. However, nonnatural zeolites have environmental issues such as increasing sodium level, removing calcium, and increasing the pH of the medium to 8.5 [22]. Hence, although natural zeolites have low efficiency in metal ion removal, they are natural with low risk in terms of environmental perspectives.

Zeolites have various applications in catalysis, agriculture, building industries, energy, and removal process of oil, ammonium, metal ions, etc. from water streams [1, 19–45]. The ratio of Si/Al_2_ in zeolite structure is used to indicate the hydrophobicity of zeolite. A higher ratio of Si/Al_2_ indicates a higher degree of hydrophobicity and lower ion-exchange capacity [70]. In addition to common applications of zeolites, another significant application of the material is for biomass immobilization as suitable carriers.

### 3.2. Zeolite-Immobilized Biomass and Its Applications

Many advantages can be offered by zeolite-immobilized biomass. According to Weiß et al. 2010 and Milán et al. 2003, zeolites were used as a support for microorganism immobilization in anaerobic reactors resulting in stabilizing and optimizing process efficiency [53, 71]. Also, the immobilized biomass obtained after the process can be used for other applications as it has a high worth. It can be considered as biomass enriched feed [15] and useful for animals [50]. In addition, Djukić-Vuković et al. 2013 reported that a simple cell separation in the fermentation area and cell reuse in repeated batch cycles were achieved by immobilization [50]. Also, Figueroa-Torres et al. 2016 reported that the acidogenic biomass immobilized in clinoptilolite is a low-cost biosorbent for copper and iron removal [48]. This is an important finding that further can make the whole process more feasible for industrial applications. There are several factors interfered in the attachment process between biomass and zeolite. As reported by MacArio et al. 2007, zeolites showed a significant property towards the immobilization process, including hydrophobic or hydrophilic behavior, acid or base character, chemical and mechanical resistance, great morphology, a total surface area, and easy water dispersion/recuperation. Also, the researchers reported that zeolites included a great number of Si-OH groups that play key roles in the adsorption process [63]. According to Djukić-Vuković et al. 2013 and Kubota et al. 2008, the surface of zeolite can adsorb biopolymers such as proteins, nucleic acids, RNA, and DNA in a selective manner as well [50, 70]. Due to these reasons, zeolites can adsorb microorganisms on their surfaces, and they can be used as an efficient [50] and potential [72] carrier in the process of biomass immobilization. On the other hand, the surface of microorganisms is full of various components, such as polysaccharides [70]. Exopolysaccharides (EPS) can have a protective role in unfavorable conditions and act as adhesives for interactions with various substrates and also as a promoter for cell aggregation and biofilm formation [50, 73]. There is a possibility that EPS may contribute to the process. According to Pazos et al. 2010 and Lameiras et al. 2008, under stress conditions in a diluted medium, the biofilm formation can increase the production of EPS, resulting in the formation of coherent biofilm with a powerful adhesion to the carrier surface [52, 54]. Djukić-Vuković et al. 2013 used* Lactobacillus rhamnosus* for immobilization onto zeolite and they reported that the bacterium produced EPS and formed a sticky layer on the surface of the microorganism; hence, it aided the immobilization process [50].  Table 3 shows various types of zeolite-immobilized biomass and their applications.

#### 3.2.1. Production of High-Value Products

The zeolite-immobilized biomass can be used for the production of some metabolites such as acids [15, 49, 50] and alcohols [46, 47]. In a research done by Shindo et al. 2001, natural zeolite abilities were compared with glass beads for the immobilization and alcohol fermentation. The results showed that the immobilization capacity and alcohol fermentation activity of natural zeolite were 2- and 1.2-fold higher than those of glass beads, respectively [46]. Also, the modified (or activated) zeolite as a biomass support can increase process yields. Weiß et al. 2010 used hemicellulolytic bacteria for the immobilization on trace metal activated zeolite and their results showed an increase of methane by 53% compared to without immobilization. The study also found that the modification of zeolite by trace metal elements such as iron, magnesium, cobalt, and nickel favors microorganisms to be grouped in small microcolonies and supply cofactors in the enzyme biosynthesis process [53]. One of the main purposes of the immobilization technique, as mentioned before, is recycling the immobilized materials. The more the number of repetitions is, the higher efficiency and low cost will be. Djukić-Vuković et al. 2016 used Mg-modified zeolite as a support for* Lactobacillus rhamnosus* immobilization and their results showed that the attachment of biomass on zeolite was strong and stable resulting in a continuous colonization during four cycles [15]. In addition, Shindo et al. 2001 used natural zeolite as a support for* Saccharomyces cerevisiae* in a bioreactor and reported that no breakage of the carrier during continuous alcohol fermentation for over 21 days was achieved [46]. Also, MacArio et al. 2007 found that the enzyme immobilized on zeolitic supports could catalyze some cycles, but it continuously leached from the support [63].

#### 3.2.2. Metal Removal

The main pollutant removal mechanisms by microorganisms include assimilation, biodegradation, and biosorption [4]. Assimilation is the process of nutrient ingestion (e.g., carbon, nitrogen, and phosphorus) from wastewater by microorganisms and using them for their growth. Biodegradation is explained as the decomposition/chemical disbanding of organic materials through microorganisms/microbial aggregates aerobically and/or anaerobically. Biosorption is a phenomenon in which microorganisms can adsorb heavy metals and organic and inorganic materials via various processes, including complexation, flocculation, ion exchange, etc. [4, 74]. There are various significant factors that interfere in the biosorption process. Figueroa-Torres et al. 2016 mentioned that there are two factors that are significantly contributing during the biomass selection as potential biosorbent, including its availability and price [48]. During the removal of pollutants, all the processes of assimilation, biodegradation, and biosorption simultaneously happen [4, 74]. In the process of metal uptake by biomass, various factors are responsible for the nature of the process and determination of the binding mechanism, including chemical groups (e.g., structural polysaccharides, amino and phosphate groups in nucleic acids, carboxyl groups in proteins. etc.) and also solubility and polarity [3, 4, 54, 74–76]. Quintelas et al. 2009 reported that the functional groups existing on the biomass (*Escherichia coli*), including carboxyl, hydroxyl, and phosphate groups, were probably the main binding sites in the biosorption process. It was also observed that the metal affinity to the biofilm pursued the arrangement of Fe(III) > Ni(II) > Cd(II) > Cr(VI). This preference may be described on the basis of metal ion electronegativity and cation/anion state [5]. In addition, various physicochemical forces such as covalent bonding, van der Waals bonding, ion exchange, and dipole/dipole interactions are other factors that can interfere in ion uptake on the adsorbent [3, 76].

As suspended biofilm alone cannot be applied especially in the continuous treatment process and also zeolite, in turn, cannot retain all the metals, there is a need to evaluate the synergetic effect between biosorption ability of biomass and retention capacity of zeolite [5]. To date, several types of microorganisms have been used for the immobilization onto zeolites in the biosorption area [5, 48, 51, 52, 54, 55, 66, 77–80]. The process efficiency of biosorption is dependent on operational parameters such as biomass concentration and pH [52]. Pazos et al. 2010 used a bioreactor, including a biofilm supported on zeolite 13X and their results revealed that the biomass concentration and pH were the key variables for the metal ion removal. Also, the process efficiency was improved by adjusting biomass concentration and pH values. They successfully reached up to 100% removal of the metal ion under the optimized conditions [52]. Using other supports along with zeolite can also be effective in increasing the process yield. Lameiras et al. 2008 used minicolumns including Granular Activated Carbon (GAC) and a natural zeolite as a support for biomass immobilization and found that the combination of GAC and zeolite columns covered with biofilm gave better performance of 42% removal than individual GAC (19%) and zeolite (18%) [54]. Another interesting issue with the system is whether the zeolite by itself is effective in the process efficiency or just acts as a support for biomass. Monge-Amaya et al. 2013 used aerobic biomass biofilm for the immobilization on untreated clinoptilolite zeolite and Energy-Dispersive X-ray Spectroscopy (EDS) analysis showed that the metal ion (copper) was not detected in the treated zeolite and only absorbed by the biomass. Therefore, they concluded that zeolite had functioned as a support for the biomass [78]. There is a prominent challenge in water stream treatment which is faced with a presence of a number of metal ions in real samples. If there is any competition between them and a special removal of the adsorbent, it is an interesting issue to discuss among researchers. Figueroa-Torres et al. 2016 reported that although the predicted amount for the maximum biosorption capacity of copper raised from 28.23 in a single system to 35.46 mg / g SSV in a binary system, the presence of both ions (copper and iron coexistence) in the medium diminished the actual biosorption capacity of biomass [48]. The mechanism of metal ion sorption by zeolite-immobilized biomass can be represented by some isotherm models, including Langmuir [5, 48], Sips [5], and Toth [5], and kinetic models such as the pseudo second-order type reaction [48, 52]. Consequently, zeolite-immobilized biomass can be considered as a promising low-cost biosorbent for metal ion removal [48].

#### 3.2.3. Nonmetal Removal

The zeolite-immobilized biomass also can be applied in the removal of nonmetals such as phosphorus and ammonium from aqueous solutions. Various factors are interfering in the cell immobilization and adsorption capacity such as carrier particle size, surface charge, and surface modification [51, 77, 79, 80]. Hrenovic et al. 2009 found that the number of immobilized cells correlated negatively with the size of support [77]. Also, Mery et al. 2012 studied the effect of zeolite particle size (0.5, 1.0 and 2.0 mm) on adherence of microorganism and adsorption capacity of ammonium, and their results showed that the greatest amount of zeolite adhered biomass was achieved for a particle size of 1 mm whereas the highest adsorption capacity of ammonium was obtained in a size of 0.5 mm [51]. As it mentioned, other factors influencing the adsorption of the microorganism in immobilization and adsorption capacity are the surface charge (defined by zeta potential) of both support and microorganism [77] and surface modification [80]. Hrenovic et al. 2009 reported that the increase of the zeta potential of supports correlated negatively with the number of immobilized cells [77]. Also, surfactant-modified zeolites (SMZ) were used as a support for Orthophosphate (p)-accumulating bacteria to remove phosphorus from wastewater. According to the results, zeolite surface modification concluded in the change of the zeta potential of particles (from negative to positive) and improvement in adsorption capacity [80].

## 4. Algal Immobilization

### 4.1. Algae

**Figure 2:**
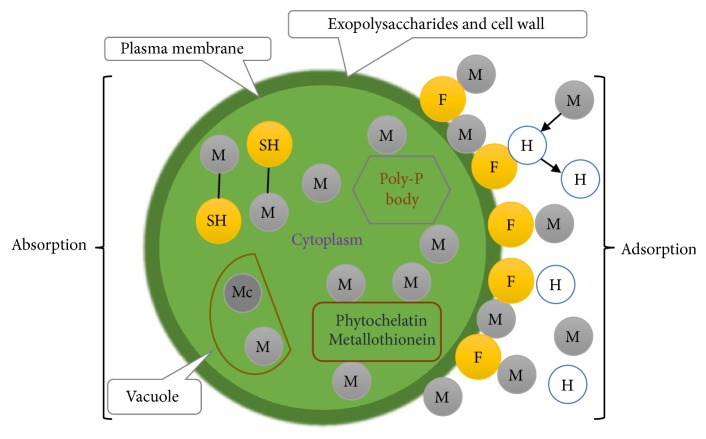
The process of metal sorption by living algal cells: M: metal ions, Mc: metal ion complex, F: functional groups, H: hydrogen ion, SH: sulphydryl (adapted from [17, 95, 99]).

One of the most famous photosynthetic organisms that are capable of living and growing in many different environmental conditions is algae [81]. Instead of using bacteria to treat wastewater, it is also possible to use algae due to the existing nutrient sources present in wastewater such as nitrates and phosphates. Algae can uptake those nutrients and use them for their cell growth [82]. Most of the researchers in the area of biotechnology prefer to work on algae because of their advantages such as producing biomass without needing many resources, increasing the level of oxygen in water effluents, reducing the level of carbon dioxide, and reaching lower operational costs [3]. Based on morphology and size, algae are classified into two groups: macro and micro. Macroalgae (or seaweed) are composed of multiple cells and divided into three groups of brown, green, and red based on their pigmentation. Microalgae are unicellular and classified into three most important groups: diatoms, green, and golden [83]. Algal biomass can be used in various applications, including production of primary and secondary metabolites for food, cosmetic and pharmaceutical industries [3, 14, 58, 84], biofuel and bioenergy [3, 14, 84–87], wastewater treatment process [3, 14, 82, 84, 88–101], animal feedstock enhancer [81], and agriculture purposes [3, 14, 94]. One of the significant applications of algae is in the bioremediation area. Various species of microalgae, including eukaryotic and prokaryotic and also their inactive forms, can be used for the bioremediation of metal ions [99]. They can be considered as suitable biosorbents due to some characteristics such as their abundance in seawater and fresh water, high metal sorption capacities, reusability, and cost-effectiveness [96]. Microalgae have been reported to have higher metal ions biosorption capacity than other microorganisms like fungi, cyanobacteria, and bacteria [3, 102, 103]. This is due to the fact that the algal cell wall has a number of functional groups such as amino (—NH_2_), hydroxyl (—OH), carboxyl (—COOH), phosphoryl (—PO_3_O_2_), sulphydryl (—SH), etc. that are mostly responsible for metal ions biosorption [82, 88, 95, 98]. As shown in Figure 2, the process of metal uptake by microalgae can be explained in two ways: the first step is rapid adsorption onto the surface of the cell, and the second step is via the absorption or intracellular uptake that is dependent on cell metabolism [82, 95]. The process of metal ion adsorption includes their binding on the cell wall, cytoplasmic membrane, capsule substances, and extracellular compounds (external polysaccharides) [13, 95] whereas the process of metal ion absorption includes binding to phytochelatins, metallothioneins, cytoplasmic ligands, etc. [95].

### 4.2. Immobilization of Algal Biomass and Its Applications

Harvesting algal biomass from water streams, either culture medium or treated wastewater, is one of the most important challenges especially in a large-scale application. Harvesting methods such as mechanical, electrical, chemical, and biological techniques are not preferable due to the high assumption of chemicals and energy [100]. Immobilization of algae can be an alternative method to overcome the issues [100]. The immobilization of algae has been started over 40 years ago [4, 59]. The advantages of immobilization are shown in Table 1. The algal immobilization can provide high-value algal biomass [3, 14] which is different than the suspended one in the content and composition of lipid/chlorophyll, cell size, surface charge, protein content, and molecular compositions [104]. Zeng et al. 2013 studied the* Chlorella *sp. immobilization within a polymeric carrier and found that the immobilized cells achieved a total lipid and chlorophyll content of 14.85% and 3.36%, respectively, which was higher than suspended ones [104]. In addition, Zeng et al. 2013 reported that although the microalgae cell growth in immobilized cultivation was slower than the suspended cells (due to their mass transfer problems), the capsule-immobilized microalgae had a systematic growth of the algal cells [105]. The similar finding was achieved by Mallick et al. 1994 who worked on the immobilization of* Anabaena doliolum* and* Chlorella vulgaris* and their results revealed that the agar-immobilized cells had a slow growth rate [106]. Furthermore, algal immobilization can increase the biosorption capacity and biomass activity as well [3]. The ability of microalgae in the attachment process depends on various factors, which are the age of microalgae whether it is in the exponential growth phase or in the stationary phase, culture state, and composition of the culture medium [13]. Furthermore, other important factors that influence the algal immobilization are the cell wall composition and surface charge of cells and carrier [107]. Eroglu et al. 2012 used chitosan nanofiber mats for the immobilization of* Chlorella vulgaris*. They reported that microalgae cell walls consisted of various polysaccharides compatible with the support surface and also* Chlorella* cells surface has a negative surface charge (related to uronic acid and/or sulfate groups) that makes an electrostatic attraction to chitosan with the positive charge (resulting from primary amine groups) [107]. In the case of immobilization support, natural biopolymers and synthetic compounds (e.g., alginate, carrageenan, agar, polyacrylamide, polypropylene, and silica gel) have been used for the algal immobilization [13, 17, 95]. Researchers often prefer to use natural polymers due to their low toxicity [17]. Among natural polymers, alginate [3, 13, 17, 102, 108] and carrageenan [3, 13] have been widely used for the algal immobilization. On the other hand, polyacrylamide [17, 109, 110] has been extensively used as a synthetic polymer due to the fact that it is more resistant than the natural polymers [17]. Also, some researchers have used other supports such as loofa sponge, chitosan, and polymer carriers [104, 106, 107, 111]. Akhtar et al. 2004 found that loofa sponge was an effective support for the entrapment of microalgae [111]. Also, Mallick et al. 1994 reported that the immobilization on chitosan was more efficient than other beads like agar, alginate, and carrageenan [106]. In addition, Eroglu et al. 2012 reported that chitosan nanofiber mat can be used as a water-insoluble and nontoxic support for algae [107]. Zeng et al. 2013 used sodium cellulose sulphate/poly-dimethyl-diallyl-ammonium chloride (NaCS-PDMDAAC) capsule for the immobilization of* Chlorella *sp. and their results showed that the immobilized cells had a robust morphology [104].  Table 4 lists various supports for algal immobilization. Immobilized microalgae can be harvested and converted to produce a variety of applications, including biofuel [3, 13, 58], removal of BOD, COD [112, 113], metal ions [14, 95, 102, 106, 108, 109, 111, 113–124], nonmetals [3, 11, 14, 106, 107, 112, 113, 119, 125–131], and use as a biosensor to measure the degree of water pollution [13, 14], toxicity of substances, and effluents in electronic devices [58] and as photosynthetic solar cells [132].  Table 4 summarizes the related researches on algal immobilization.

#### 4.2.1. Production of High-Value Metabolites

Immobilized algae can be applied to the production of valuable metabolites. Desmet et al. 2015 synthesized a robust (Ca-alginate-SiO_2_-polycation) shell: (Na-alginate-SiO_2_) core hybrid system for use in an encapsulation of* Dunaliella tertiolecta*. They reported that high photosynthetic activity was achieved over a long time (more than one year). The system can be applied in harvesting high-value compounds, producing biofuel cells to photosynthetic solar cells for production of electricity, energy transformation, etc. [132].

#### 4.2.2. Removal of Nitrate and Phosphate

Nutrient removal, including nitrate and phosphate, is another application of immobilized algae [11, 107]. Eroglu et al. 2012 used chitosan nanofiber mats immobilized* Chlorella vulgaris* to remove nitrate from liquid effluents. They reported that around 30 % of initial nitrate value was diminished within the first two days by chitosan uptake (physicochemical adsorption) and, after that, the remaining nitrate uptake continued with the growth of algae using it for its cellular metabolism. Their results also showed the overall removal rate of 32 ± 3% for* Chlorella*-absent and 87 ± 4% for* Chlorella*-attached chitosan mats [107]. Zeng et al. 2012 used* Chlorella *sp. and entrapped the cells in a polymer carrier of sodium cellulose sulphate/poly-dimethyl-diallyl-ammonium chloride (NaCS-PDMDAAC) to make algal capsules for the removal of total nitrogen (T-N) and phosphate (PO_4_^3−^ -P) from artificial wastewater. Their results showed that high removal rate of 12.56 mg/g biomass per day for T-N and 10.24 mg/g biomass per day for PO_4_^3−^ - P was achieved at the end of the treatment process [11].

#### 4.2.3. Removal of Metal Ions

The amount of metal ion uptake in suspended and immobilized algal systems is different. On the one hand, the biosorption capacity of immobilized systems is higher than the suspended one and on the other hand, the number of repetitions in immobilized cells makes the researchers shift from the suspended to the immobilized one in the removal of metal ions [111, 117, 124]. Nasreen et al. 2008 used* Chlorella sorokiniana* for immobilization in loofa sponge as a biosorbent for the removal of Cr(III) from an aqueous solution. Their results showed a higher increase in uptake of chromium (17.79%) for the immobilized biomass than the suspended one [124]. Akhtar et al. 2004 applied the same immobilized system for the removal of nickel(II) and their result revealed a significant increase of 25.3% in biosorption capacity compared to the suspended one [111]. Furthermore, Nasreen et al. 2008 and Akhtar et al. 2004 and 2003 reported that this immobilization system showed an excellent physicochemical stability without any significant release of algae in nickel(II) removal and it has a potential to be used for 5 and 7 cycles with a little decrease in metal uptake capacity [111, 117, 124]. According to Wan Maznah et al. 2012 and Bayramoglu and Arıca 2009, the main mechanism in the biosorption process of immobilized microalgae is an adsorption in the form of physical and ion exchange interactions [108, 123]. In the biosorption process of immobilized microalgae, some operational parameters interfere and give an impact to the biosorption capacity, including pH, biosorbent concentration, the initial concentration of metal ions, time, and temperature [102, 108, 111, 119, 123, 124]. The pH of the solution is one of the most important factors in biosorption as it affects the metal ion speciation and the surface charge of the hybrid biosorbent [108]. According to researchers, the range of pH for the maximum biosorption was around 4 to 8.0 [102, 108, 111, 119, 124]. The low metal ion biosorption at low amounts of pH is related to the competition between metal ions and protons for binding sites and at a high amount of pH is related to the decreased solubility of metal ions [124]. The biosorbent concentration is another significant factor affecting biosorption capacity. Akhtar et al. 2004 used* Chlorella sorokiniana* for immobilization on loofa sponge for the removal of nickel(II). They reached an increase in metal ion uptake with an increase in immobilized biomass concentration up to 2.5 g/L [111]. As a matter of fact, cell immobilization permits little or no interaction with the rest of immobilized cells and does not let cells clump; hence, the binding sites available on the cell wall have maximum accessibility to metal ions [111]. According to the findings of researchers, initial metal ion concentration is another operational parameter that can have an effect on the growth of algae, cell count, chlorophyll content, and adsorption capacity [123]. Wan Maznah et al. 2012 used* Chlorella *sp. and* Chlamydomonas *sp. as biomass for the immobilization in sodium alginate beads to remove copper and zinc. The results of the biosorption capacity of* Chlorella *sp. of 33.4 and 28.5 mg/g were achieved after 6 hours for copper and zinc, respectively. They reported that metal ions affected the growth of algae at a concentration of 5 mg/L leading to significant diminishment in the cell count and contents of chlorophyll a and b for microalgae [123]. In the case of metal ion removal by immobilized microalgae, many researchers reported that the adsorption can improve when the initial metal ions concentration increases up to 200, 500, or 600 mg/L in the medium [102, 108, 111]. Also, time and temperature are other factors during algal immobilization. A study conducted by the researchers showed that the kinetics of metal ion biosorption by immobilized cells was rapid in the first 5 min [117, 124] and can reach up to an equilibrium in 15 or 60 min [102, 111, 117, 124] and after that available sites on biosorbent were a limiting factor [124]. Bayramoğlu et al. 2006 considered the temperature range from 5 to 40°C in biosorption process but they did not observe any effect on the adsorption capacity of immobilized cells (*Chlamydomonas reinhardtii* in Ca-alginate), and they reported that an adsorption of metal ions was temperature independent [102]. In case of immobilized algae, the biosorption equilibrium can be represented by Langmuir [102, 108, 111, 124], Freundlich [102, 108, 111], and Dubinin-Radushkevich isotherm [108] models and the biosorption kinetics follow the second [108] and pseudo-second [124] order kinetic models.

## 5. The Potential Application of Zeolite as an Ideal Carrier for Algal Immobilization

One of the most significant drawbacks in algal immobilization is finding a suitable carrier. Natural carriers such as carrageenan and alginate have the problem of low stability [13] and synthetic polymers such as polyacrylamide face the drawbacks of high cost and toxicity to living cells [17]. Although the researchers have tried many materials as a support such as loofa sponge, chitosan, and polymer carriers [104, 106, 107, 111], many works need to be done to prove its feasibility. The ideal carrier for the algal immobilization must fulfill some requirements such as nontoxicity, cheapness, acceptable process efficiency, being potential to reuse, and stability. However, it seems that reaching these aims and finding an excellent carrier with these characteristics need further researches and remain a challenge. One of the materials as a suitable candidate for algal immobilization is zeolite. As mentioned in this paper, to date, various types of zeolite have been successfully used for the immobilization of bacteria and yeast. If there is any possibility to apply them as a carrier for the immobilization of different types of algae, it needs to be researched. In case of successful immobilization, one of the most important applications of the bioparticle (i.e., zeolite-immobilized algae) can be in the biosorption process of metal ions in which zeolite acts as a carrier and probably contributes to the sorption process resulting in improved efficiency. All these possibilities need to be studied and justified in the future.

## Figures and Tables

**Table 1:** Advantages of cell immobilization over suspended cell.

Advantages of immobilization	References
Simplifying biomass harvesting	[3, 4, 13]
Higher cell density	[3, 4]
Enhancing operational stability	[3, 4]
Avoiding cell washouts	[3]
Increasing cell resistance to unfavorable factors (temperature, acidity, and toxic compounds)	[13]
Occupying less space	[3]
Easier to handle	[3]
Using repeatedly	[3, 14]

**Table 2:** Immobilization methods of microorganisms and their advantages and disadvantages (Figures adapted from [4, 62]).

Method	Definition	Advantages	Disadvantages	Figure	References
Adsorption	Based on physical interaction between biomass and support surface	(i) Simplicity (ii) Minor influence on biocatalyst conformation (iii) No need for chemicals utilization	(i) Relative weakness of the adsorptive binding forces	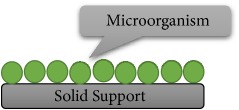	[4, 18, 50, 62]

Covalent bonding	Based on bonding reaction of some groups (e.g. —COOH or —NH_2_) at the surface of microorganism	(i) More stable than adsorption	(i) Involves toxic bifunctional reagents (ii) Decreasing bioactivity of microorganism	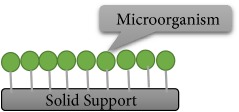	[4, 13, 18, 62, 63]

Cross-linking	Based on linking bio-macromolecules to each other by covalent bonds using multifunctional reagents (e.g. glutaraldehyde)	(i) Simple method	(i) Very difficult to control	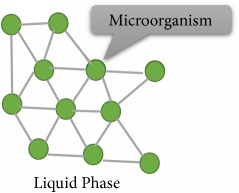	[4, 18, 62]

Encapsulation	Based on encapsulating microorganisms into synthetic or natural polymers	(i) Natural polymers (ii) Synthetic polymers	(i) Diffusion limitation	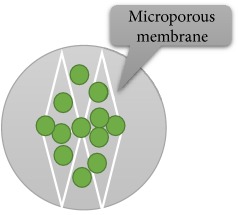	[4, 18, 62]

Entrapment in a matrix	Based on microorganism entrapment in porous polymer support	(i) Natural polymers (Higher nutrient/product diffusion rates and more environmentally friendly) (ii) Synthetic polymers (More stable)	(i) Transfer limitations	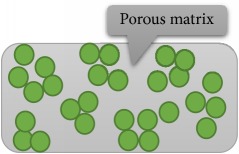	[3, 4, 18, 59, 61, 62]

**Table 3:** Various types of zeolite-immobilized biomass and their applications.

Type of zeolite	Immobilized biomass	Applications	Findings	References
Clinoptilolite	*Acidogenic biomass (anaerobic sludge)*	Copper and iron removal	Biosorption capacity of 28.65 mg/g copper and 34.72 mg/g iron	[48]
Mg-modified zeolite	*Lactobacillus rhamnosus*	L (+) lactic acid production	Overall productivity of 1.41 g/L/h	[15]
Zeolite	*Actinobacillus succinogenes*	Succinic acid production	Productivity of 2.83 g/L h	[49]
Zeolite (type 13X)	*Lactobacillus rhamnosus*	Lactic acid production	Productivity of 1.69 g/ L	[50]
Chilean natural zeolite	*Bacterial microorganism*	Ammonium removal	Adsorption capacity of 2.7 mg/g	[51]
Zeolite 13X	*Arthrobacter viscosus*	Chromium removal	100% removal	[52]
Trace metal activated zeolite	*Hemicellulolytic bacteria*	Biogas production	Methane increase by 53%	[53]
Zeolite NaY	*Escherichia coli*	Chromium, cadmium, iron, nickel removal	100% removal for iron	[5]
Natural zeolite	*Arthrobacter viscosus*	Chromium removal	18% removal	[54]
Natural zeolite	*Anaerobic microorganisms*	Anaerobic treatment of Wastewater	COD removal efficiencies as high as 90%	[55]
Natural zeolite	*Saccharomyces cerevisiae*	Ethanol fermentation	Ethanol concentration 2% (w/v)	[46]
Zeolite	*Saccharomyces cerevisiae*	Alcoholic fermentation	Productivity of 35.6 g ethanol/L.h	[47]

**Table 4:** Various types of immobilized microalgae and their applications.

Microalgae strain	Kind of support	Applications	Findings	References
*Chlorella vulgaris*	Chitosan nanofiber mats	Nitrate removal	Around 87% removal	[107]
*Chlorella sp.*	Sodium alginate beads	Copper and zinc removal	Biosorption capacity of 33.4 for copper and 28.5 mg/g for zinc	[123]
*Chlorella sp.*	NaCS-PDMDAAC	Total nitrogen (T-N) and phosphate (PO_4_^3−^ -P)	Removal rate of 12.56 for T-N and 10.24 mg/g biomass per day for PO_4_^3−^ -P	[11]
*Scenedesmus quadricauda*	Ca-alginate beads	Removal of Cu(II), Zn(II) and Ni(II)	Maximum adsorption capacity of 75.6, 55.2 and 30.4 mg/g for Cu(II), Zn(II) and Ni(II)	[108]
*Scenedesmus sp.*	Chitosan	Removal of nitrate and phosphate	70% nitrate and 94% phosphate removal	[131]
*Chlorella sorokiniana*	Loofa sponge	Removal of Cr(III)	Maximum biosorption capacity of 69.26 mg Cr(III)/g biosorbent	[124]
*Chlorella vulgaris* and *Scenedesmus rubescens*	The twin-layer system	Removal of nitrogen and phosphorus	Removal to less than 10% of the initial concentration	[125]
*Tetraselmis chui*	Calcium alginate	Removal of Cu and Cd	Removal of all Cu and removal of around 20% of total Cd	[115]
*Scenedesmus intermedius Chod. *and* Nannochloris sp.*	Calcium alginate beads	Phosphorus (P) and nitrogen (N) uptake	0.012 mg P h^−1^ and 0.009 mg N h^−1^ for Scenedesmus intermedius and 0.009 mg P h^−1^and 0.006 mg N h^−1^ for Nannochloris sp.	[127]
*Chlorella sorokiniana*	Loofa sponge	The removal of nickel(II)	Biosorption capacity of 60.38 mg nickel(II)/g	[111]
*Trentepohlia aurea*	Filter paper	Removal of inorganic nitrogen sources	The removal rate of total inorganic N ion of 5.11 mg N l^−1^day^−1^	[128]
*Chlorella sorokiniana*	Vegetable sponge of Luffa cylindrica	Cadmium removal	The cadmium sorption capacity of 192 mg/g	[116]
*Chlorella sorokiniana*	Luffa sponge discs	Removal of Ni(II)	The removal of 97% of equilibrium loading	[117]
*Chlorella vulgaris*	Calcium alginate beads	Removal of N and P	100% removal of NH_4_^+^-N and around 95% reduction of PO_4_^3−^-P	[129]
*Chlorella homosphaera*	Sodium alginate	Biosorption of cadmium, zinc and gold	Near 100% metal removal for cadmium and zinc and 90% removal of gold	[121]
